# Synchronized Renal Blood Flow Dynamics Mapped with Wavelet Analysis of Laser Speckle Flowmetry Data

**DOI:** 10.1371/journal.pone.0105879

**Published:** 2014-09-12

**Authors:** Alexey R. Brazhe, Donald J. Marsh, Niels-Henrik Holstein-Rathlou, Olga Sosnovtseva

**Affiliations:** 1 Department of Biophysics, Biological Faculty, Moscow State University, Moscow, Russia; 2 Department of Molecular Pharmacology, Physiology, and Biotechnology, Brown University, Providence, Rhode Island, United States of America; 3 Department of Biomedical Sciences, Panum Institute, University of Copenhagen, Copenhagen, Denmark; Max-Delbrück Center for Molecular Medicine (MDC), Germany

## Abstract

Full-field laser speckle microscopy provides real-time imaging of superficial blood flow rate. Here we apply continuous wavelet transform to time series of speckle-estimated blood flow from each pixel of the images to map synchronous patterns in instantaneous frequency and phase on the surface of rat kidneys. The regulatory mechanism in the renal microcirculation generates oscillations in arterial blood flow at several characteristic frequencies. Our approach to laser speckle image processing allows detection of frequency and phase entrainments, visualization of their patterns, and estimation of the extent of synchronization in renal cortex dynamics.

## Introduction

Laser speckle imaging complements other blood flow measurement techniques, such as laser Doppler flowmetry [1] and magnetic resonance imaging [2] and allows to measure local blood flow distribution with a relatively high spatial-temporal resolution and does so without the need for emission scanning [3]. Laser speckle imaging instrumentation can also be made radically low-cost, allowing for wide educational or mobile applications [4].

Speckle is an interference pattern produced by coherent (laser) light scattering on a rough surface. The intensity of speckle patterns fluctuates if the illuminated object contains individual moving scatterers such as blood cells. These fluctuations blur the speckles, leading to a reduction of the local speckle contrast, with the contrast value inversely proportional to the flow speed. These principles form the basis of laser speckle flowmetry (LSF) [3], [5].

Speckle imaging techniques have been used to monitor blood flow velocity in a number of tissues [6]. This method has been applied to the retina [7], skin [8], mesenteric microcirculation [9], and during focal ischaemia and cortical spreading depression (CSD) in the brain [10]. Most studies address steady state tissue blood perfusion rather than temporal changes associated with different regulatory mechanisms. The full-field speckle technique performs imaging of instantaneous blood perfusion in real time and simultaneously from different points within a field, and is therefore a promising tool for detecting and measuring synchronous patterns of many operating units involved in local blood flow regulation.

Nephrons produce oscillations of proximal tubule hydrostatic pressure, renal tubular flow rate, and chloride concentrations with a period of 30–50 sec caused by tubulo-glomerular feedback (TGF) [11], [12], a negative feedback mechanism that transmits signals from a nephron sensing site to the arteriole supplying that nephron with blood. Changes in the feedback signal induce changes in cytoplasmic calcium concentration and membrane electrical potential in smooth muscle cells of the arteriole. The arterioles are electrically conductive, providing nephrons an opportunity to interact by exchanging electrical signals, hemodynamic coupling, or both. To estimate the number of nephrons that form synchronized clusters, and to assess the factors that modify cluster size, one needs a method for simultaneous and continuous measurements of dynamical phenomena in blood flow speed in many nephrons, a problem set for which laser speckle microscopy is well suited. We applied this method to detect changes in many nephrons on the kidney surface of anesthetized rats [13]. Different oscillatory components in the kidney perfusion have been also explored by Scully et al. [14].

The classical concept of synchronization [15], [16] considers the interaction of two or more oscillators, each with their own sources of energy, the coupling causing an adjustment of the time scales in the form of frequency and phase entrainments. Local coupling typically produces waves or pulses that propagate across the interacting units [17]. Phenomena associated with global coupling structure are global synchronization and various forms of clustering in which the ensemble splits into subgroups of synchronized oscillators, but such that each subgroup maintains its own dynamics [18]. Synchronization theory has been widely applied to the analysis of multivariate biological signals. Rosenblum et al. [19] discussed how the phases and frequencies can be estimated from time series and techniques for detection and quantification of synchronization from biomedical data. Wavelet-based tools to study the dynamics of biological processes have been widely applied [20].

Experimental studies of nephron synchronization have thus far been limited to measurements on pairs or triplets of nephrons [21]–[23]. We have previously used the laser speckle technique to measure nephron blood flow on the kidney surface of aneshetized rats [13], but not all nephrons in the field could be sampled, so that not all clusters could be identified. Here we develop a more extensive sampling technique, making it possible to study local and global interactions between rhythmic processes, using the kidney to illustrate the patterns and clusters of frequency and phase entrainments.

## Results

### Fourier-based analysis

**Figure 1:**
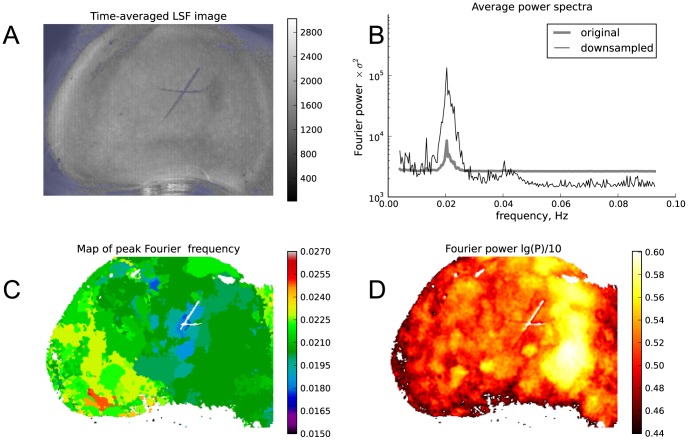
Fourier representation of the TGF oscillations. (A) Time-averaged LSF image of a rat kidney. Blue regions are masked and excluded from analysis. (B) Fourier power spectra averaged over all the non-masked pixels of the original (gray) and spatially downsampled (black) data. (C) Location of the main Fourier peak in the TGF band mapped across a kidney surface. (D) Log peak intensities of Fourier power spectrum mapped across the kidney surface. Time series from each pixel were normalized to their standard deviation.

To determine whether the TGF rhythm could be detected in our LSF data we first applied Fourier analysis to image series obtained from the ventral surface of a rat's kidney. Non-renal tissue was masked, as described in Methods section. Representative results are summarized in Figure 1.

We applied fast Fourier transform (FFT) to the LSF temporal signals (normalized to the signal standard deviation) from each pixel, squared the result, and clipped the spectra to a frequency range around the TGF band. The mean power spectrum averaged over all the pixels in the kidney showed a pronounced peak near 0.02 Hz, indicating the presence of oscillations with similar frequencies in many regions of the kidney surface (Figure 1D). To facilitate spatial analysis we downsampled each frame from the original 760×568 pixels to 380×284 pixels as described in Methods section. This procedure had the additional benefit of enhancing the signal-to-noise ratio because of the spatial smoothing prior to resizing. The average Fourier power spectrum for the spatially resized data, shown in Figure 1D with a black line, shows an even stronger and more detailed peak around 0.02 Hz. All subsequent analyses of the LSF data were done on the spatially resized recordings.

A map of the base 10 logarithm of the peak amplitude of the power spectrum within the TGF band is shown in Figure 1B. The map was calculated from LSF time series in each pixel of the spatially downsampled images. The resulting image shows uneven distribution of the peak amplitudes. A similar mapping, this time using the locations of the TGF peak, is shown in Figure 1C. Domains with similar frequencies immediately stand out as areas with the same color. Areas with the highest peak power (B) are also the most uniform in frequency (C).

**Figure 2:**
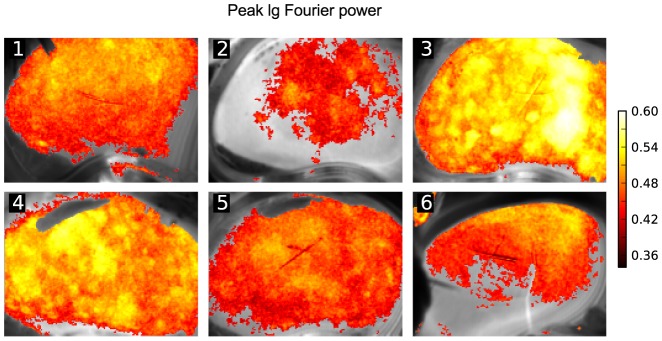
Mapping intensity of TGF oscillations in 6 animals. Grayscale images show LSF frames, pseudo-colors represent the log peak power of the Fourier spectrum in the TGF band 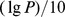. Time series from each pixel were normalized to their standard deviation. Areas where the 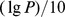 value was lower than 95%-confidence interval, estimated from Monte-Carlo simulations, are transparent.

Because of the normalization, the values in Figure 1 B are given in units of the variance of the signal and thus reflect the signal-to-noise ratio with respect to TGF oscillations. This allowed us to set empirical thresholds to separate pixels with a reliable TGF rhythm from others. Threshold was determined in a Monte-Carlo simulation as the 95% percentile of the base 10 logarithm of the maximum value in a Fourier power spectrum of 10^6^ random white noise signals. We did the same mapping as in Figure 1 B for 5 other kidney preparations and found that substantial and continuous areas displayed significant TGF oscillations in most, but not all preparations (Figure 2). The preparation under number 3 in Figure 2 is the same as in Figure 1. Only pixels with significantly high Fourier power in the TGF band were used in the wavelet-based analysis of frequency and phase synchronization.

### Wavelet-based analysis

Because the Fourier analysis showed periodic behavior in the LSF data, we sought ways to elicit possible patterns of spatial synchronization across the kidney surface. To this end we used the continuous wavelet transform (CWT). We applied the transform to the normalized time series from each pixel, and then determined whether spatial or temporal patterns emerged in the distribution of dominant rhythm frequencies and instantaneous phases.

**Figure 3:**
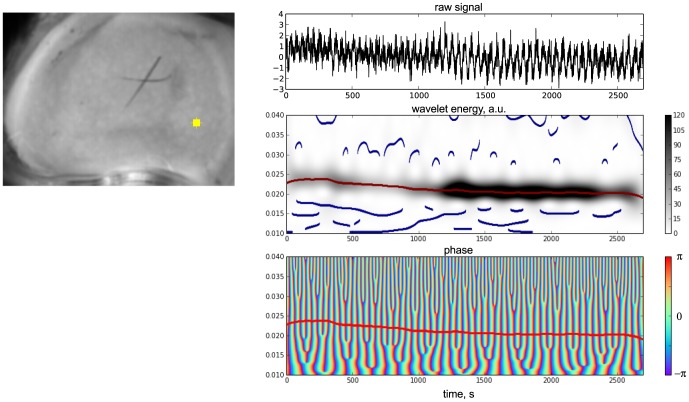
CWT-based identification of the instantaneous frequency and phase of the dominant rhythm for an example pixel in a LSF data (the same preparations as in Figure 1). (A) time-averaged LSF frame. (B) Top, blood flow signal extracted from the yellow mark in (A) and normalized to its standard deviation; middle: wavelet spectrogram of the normalized signal, ridges of wavelet modulus maxima (blue) and the dominant ridge (red); bottom: time-frequency representation of the wavelet phase, the dominant rhythm is show with the red line (same as above). (C) Map of the mean frequencies of the main rhythm, identified in each pixel of the kidney image.

A normalized LSF signal and its corresponding wavelet spectrogram from an example pixel location on a kidney surface (marked with in yellow in Figure 3A) are shown in the upper and middle panels of Figure 3B.

The middle panel of Figure 3B also illustrates the automated identification of the dominant rhythm from the wavelet spectrogram. This was done as follows: we identified local maxima of instantaneous wavelet power spectra for each time point and then found and labeled contiguous “ridges” of such local maxima (shown as blue and red lines). Each ridge was assigned with a score defined as a product of the ridge time span and the averaged wavelet power skimmed by the ridge. The dominant frequency at a given time point was then defined as the frequency of the “winner” ridge (shown in red) with the highest score at the given time point. We will use the terms dominant wavelet ridge and dominant rhythm interchangeably.

Besides wavelet spectrograms, we calculated wavelet phasegrams of the signal defined for a complex-valued wavelet coefficients 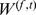 at time *t* and frequency *f* as: 
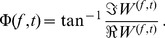
(1)


This resulted in a 2D array of wrapped phase values from 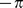 to 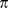 (Figure 3B, lower panel). Instantaneous phase values corresponding to the dominant rhythm were extracted as phase values at the frequency of the dominant ridge, defined above.

**Figure 4:**
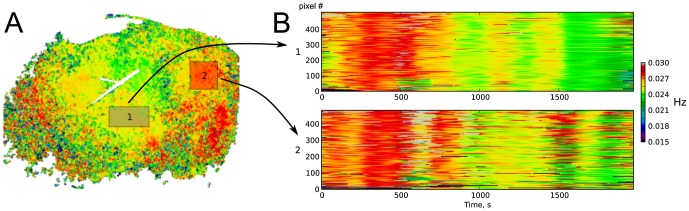
Collective changes in the dominant rhythm frequency (preparation 4 shown in Figure 2). (A) Map of time-averaged frequencies of the dominant rhythm across the kidney surface. (B) Time dynamics of the dominant rhythm frequency in each pixel within square areas in (A), after re-ordering to a 1D line. Frequency is shown as color. Changes in frequency tend to coincide in populations of pixels.

A pixel-to-pixel mapping of time-averaged frequencies of the dominant rhythms is shown in Figure 3C. The distribution of dominant frequencies across the kidney surface is comparable to the one obtained from Fourier mapping (Figure 1C. Pixels with similar average frequencies of dominant rhythms tended to aggregate in spatially contiguous areas (Figures 3 C, 4 A.) Moreover, temporal swings of the instantaneous frequencies of dominant rhythms occurred in groups of neighboring pixels in a coordinated manner, which is illustrated in Figure 4 B. Thus, the wavelet rhythm mapping suggests that pixels with close frequencies are not randomly scattered across the kidney surface but are organized in locally linked areas.

### Phase dynamics

**Figure 5:**
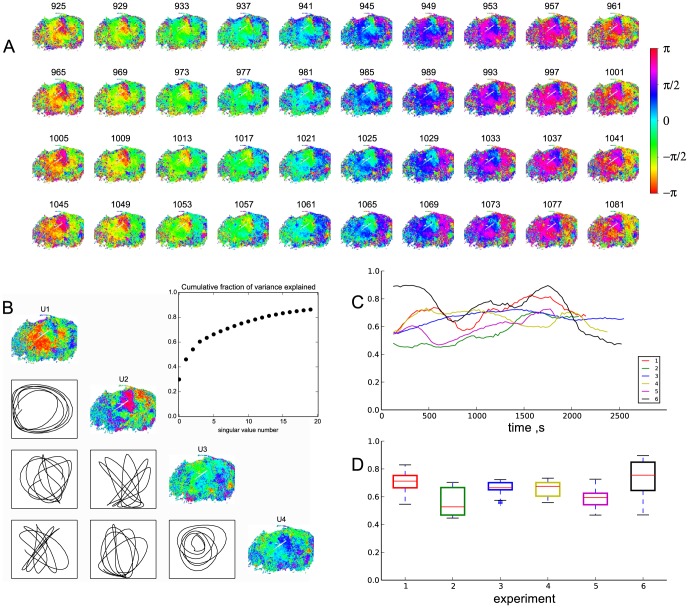
Organized spatial phase dynamics. (A) Phase maps for a short time interval (the same preparation as in Figure 4), every 4th frame is shown. Wrapped phase is color-coded from 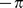 to 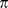. Each row of 10 frames roughly corresponds to one period of TGF oscillation. Maps in each column tend to reproduce their phase pattern for 4 periods. (B) First four empirical spatial eigenmodes (PC1–PC4) for the time period from 900 to 1100 seconds and the corresponding paired phase plots, displaying how coefficients for the pairs of modes change with time in relation to another mode. Clear oscillatory behaviour is seen for the first two eigenmodes, which correspond to concentric phase wave-like pattern. (inset) Cumulative fraction of variance explained by the first 20 modes; first two modes capture around 45% of the variation in the data. (C) Dynamics of the fraction of variance explained by the first 4 modes in moving 250 s-wide time windows for the 6 experiments shown in Figure 2. (D) boxplots that summarize the values shown in (C), phase delays are highly spatially organized over the whole experiment.

We next analyzed the spatio-temporal behavior of the wavelet phase maps defined in (1). Spatial organization of the wavelet phase dynamics displayed collective modes. An example of phase dynamics is given in Figure 5A. Every 4th frame is shown for the frames from 925 to 1081 (2.5 minutes) of the same recording as shown in Figure 4. This timespan roughly encompasses four periods of the TGF rhythm, and each row approximately represents one period. The frames in each column of the figure repeat with regard to the previous row, indicating stability of spatial organization of the phase lags during this time interval. It is therefore apparent that phase dynamics is cooperative in the synchronized regions and is organized in near-concentric spatial patterns.

The level of spatio-temporal correlation between phase dynamics in individual pixels can be tested with singular value decomposition (SVD) [24], [25] which provides empirical normal modes of the observed data. The more dynamics of the data can be captured by the first few empirical normal modes, the more organized is the dynamics [26], [27]. During approximately the same time interval as shown in Figure 5A, (900 to 1100 seconds), the first two spatial eigenmodes explained about 45% of observed variance. Their corresponding weights when plotted one vs the other displayed a clear oscillatory behavior, indicating a repeated switching between the two modes, while the next two modes described another 15% of observed variance together and also showed a coupled dynamics of phase trajectories (Figure 5B). Thus, dynamics of the phase lags displayed a concentric wave-like patterns on the kidney surface. This observation was corroborated by the oscillatory switching between the two eigen-modes of the phase-lag movies. These results suggest a high level of correlation in the dynamics of phase in a large population of pixels.

It was interesting to test if this collective phase behaviour was present at other moments of time and whether it was common in other experiments. Figure 5 C shows how the fraction of variance explained by the first four eigenmodes defined for a sliding 250 s-wide window of (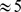 periods of TGF) changes at each moment of time for the 6 preparations shown in Figure 2. On average, the first four modes explained 65.6% of variance in the data. In 99% of time this value stayed between 45.2% and 89.5% with half of the data above 66.6% variance explained (Figure 2 D).

In experimental studies, where time series are of limited length and noisy, one quantifies the degree of interrelation between two signals *k* and *l* by means of phase coherence (PC) index [15], [19], [28], also termed as synchronization index: 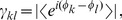
(2)


where 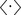 denotes averaging in time, 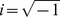, and 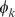 is the unwrapped (monotonically increasing) phase of the signal *k*. The index is close to 1 in the case of the pairwise phase locking and zero otherwise.

**Figure 6:**
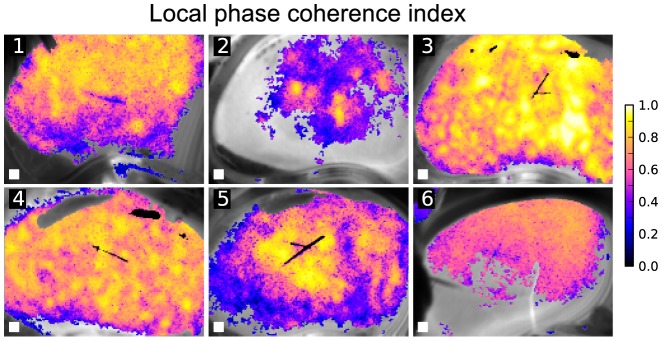
Local coherence maps for the preparations shown in Figure 2. Areas of highly locally coherent dynamics can be seen in all preparations, even in the one where only small fraction of the kidney surface showed significant TGF peak in Fourier spectrum. White square in the lower left corner show the span of the neighborhood area.

This synchronization measure is defined for two signals while imaging aplication necessitates quantification of spatial interrelations among many signals in areas of pixels. Total pairwise pixel-to-pixel calculation of the PC index was computationally inconvenient due to the large number of pixels in our images. Instead, for each pixel we calculated the PC index between phase dynamics in this pixel and all other pixels in a neighborhood of this pixel and averaged the resulting PC indices. This produced a mapping of how synchronized each pixel in the kidney was with regard to its neighborhood and whether its phase dynamics was supported by neighboring pixels. The size of the neighborhood was chosen to match nephron density on the kidney surface. In our setup, median nephron-to-nephron spacing on the kidney surface was approximately 13.5 pixels with 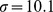 pixels, as estimated from Delauney triangulation of the star vessel positions visualized as described in [13]. The resulting local coherence maps for a 8-pixel neighborhood are shown in Figure 6. In most of the experiments we were able to observe substantial areas of locally synchronized TGF oscillations. Even though in some preparations (e.g. number 2) the Fourier-derived masks labeled only relatively small areas as having significant TGF oscillations, these areas varied in their local phase coherence from low to very high. In some preparations (e.g. 3) almost the whole surface of the kidney was locally synchronized, while in other (for example 2 and 5) there were islets of high local synchronization surrounded by less synchronized periphery.

**Figure 7:**
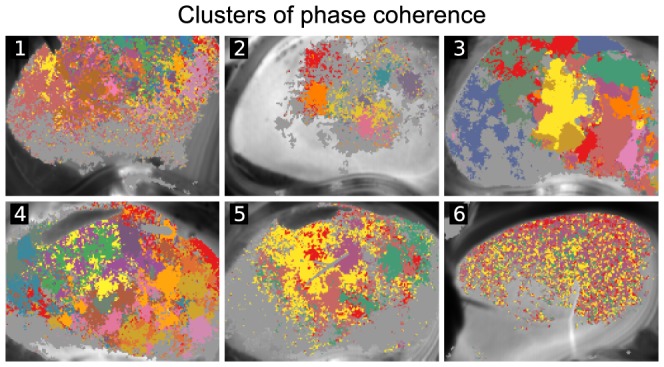
Clusters of coherent pixels. Taking peaks of local coherence maps as tentative cluster centers, each pixel was affiliated according to the center it was most coherent with. Different clusters are shown in different colors. If phase coherence of a pixel was less then 0.5 with any of the cluster centers, it was considered unclustered (shown as gray).

We next tried to find clusters of the coherent phase dynamics to uncover spatial ordering of the areas of synchronized TFG blood flow oscillations. Noting that local maxima of the local coherence maps mark pixels which are most coherent with their neighborhood and thus can serve as potential cluster centers. PC indices can thus be computed for phase dynamics in any pixel *k* of a recording and each pixel *m* from the set of local maxima from the corresponding local coherence map. Cluster affiliation of the pixel *k* is then chosen with such tentative cluster center *m* that maximizes their PC index 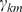. To allow for some pixels not to belong to any clusters, we labeled a pixel *k* unclustered if it didn't have 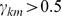 with any cluster center *m*. The results of such clustering approach are shown in Figure 7. It becomes evident that areas with high local coherence segregate into relatively contiguous clusters of synchronization whereas areas with low local coherence tend to be unclustered (shown in gray). The number of clusters and their contiguity varied from preparation to preparation, but the qualitative picture of kidney clustering remained similar. With white squares in Figure 6 approximately matching nephron to nephron spacing, it is clear that clusters e.g. in preparation 3 could embrace several or many nephrons and in general were larger than single surface nephrons.

## Discussion

Previously we performed modeling studies of the spatial dynamics of a nephro-vascular network consisting of individual nephrons connected via a tree-like vascular branching structure [29], [30]. Postnov et al. demonstrated that the nearest nephrons in a binary tree structure are synchronized in-phase due to a vascular propagated electrical coupling; the next few branching levels display a formation of phase-shifted patterns due to hemodynamic coupling and distantly located areas show asynchronous behavior [30]. Marsh et al. examined how the network structure affects nephron synchronization: the symmetric model achieved synchronization of all functional units while 1% variation in nephron length caused extensive desynchronization, although synchronization was maintained in small nephron clusters [29]. The effects of variation in vascular anatomy remain to be studied.

We devised a framework to analyse rhythmic activity and synchronization patterns in laser speckle flow imaging data or in general in any time-lapse imaging data with rhythmic temporal components. In the rat kidney data we analyzed the TGF-driven oscillations in superficial blood flow. We mapped TGF rhythms across the whole kidney surface and identified areas of spatially synchronized TGF oscillations. Approximately 2/3 to 3/4 of a kidney's nephrons are of a structure and length similar to those found on the surface, and it seems reasonable to assume all of nephrons of this group will have similar patterns of interaction and synchronization. The remaining nephrons are longer, the length variation is not normally distributed, and there have been no measurements of their dynamics. Speculation at this point is unwarranted.

Fourier analysis of the dynamical LSF imaging data confirmed the existence of blood flow oscillations in the TGF sub-band (Figure 1). We incorporated the Fourier-based analysis in the pipeline of our method to define areas with significant TGF oscillations in the LSF data (Figure 2). Once significant pixels are identified, we perform CWT on the LSF time courses in each significant pixel and obtain instantaneous frequency and wrapped phase values (Figure 3). As suggested by Figure 3 C and Figure 4 average TGF frequencies tended to make spatial clusters, and changes in instantaneous frequencies were coordinated among neighboring pixels. This led us to a more detailed analysis of phase relationships between different pixels.

Visual inspection of the calculated phase movies immediately suggested spatial structuring in the phase lags between the pixels. Changes in the wrapped phase appeared as nearly-concentric phase lag patterns (Figure 5 A). This was supported by SVD analysis of the phase data: first two empirical eigenmodes could explain about half of the variation in the data, and the alternation between their corresponding weights indicated oscillatory behavior (Figure 5 B). These two images show relatively short excerpts from the original recording. Spatial correlation at other times and in other preparations was illustrated by plotting the ratio of variance in the data explained by the first four SVD components within a sliding 250 s window. Though this measure could drift over time, it remained relatively stable and didn't differ much from animal to animal, including recordings where the area with significant TGF oscillations was scarse (e.g. recording 2).

With further analysis of the phase coherence index, an established measure of synchronization, we showed that the kidney surface varied with regard to how well a given pixel was synchronized with its neighborhood (Figure 6). We then used locations of peak local synchronization as seeds for a clustering algorithm and obtained cluster maps of mutually synchronized areas (Figure 7). It is clear that clusters tend to be spatially dense and structured, except for the experiment 6, where cluster affiliations appeared more intermixed than in the other recordings.

Physiologically, both spatial phase lags between pixels and clustered organization of the synchronized areas could be important to avoid macroscopic blood pressure oscillations, resulting from synchronized blood flow oscillations in the majority of nephrons. Currently we report a framework to analyze synchronization phenomena in the kidney blood flow, and it calls for future studies targeted at the ways to enhance or destroy the synchronization between the nephrons, whether chronically or acutely.

## Methods

### Ethics

All experimental protocols were approved by the Danish National Animal Experiments Inspectorate.

### Laser speckle flowmetry imaging

Experiments were performed on male Long Evans rats, body weight 275–325 g, purchased from Taconic (Lille Skensved, Denmark). The animals were anesthetized with sevoflurane. The experimental protocol is described in detail in Ref. [13]. We used 6 animals in this study, in each one recording was made. Figures 2, 5 (C,D), 6,7 thus encompass all data and illustrate the variability range among different animals.

Physical principles and the algorithm for laser speckle contrast (LSC) and flowmetry imaging are reviewed e.g. in Ref. [31]. In short, laser light, illuminating a rough surface creates a stochastic interference pattern, termed speckles. Moving scatterers, such as red blood cells in the vessels, blur the speckle pattern integrated by a camera over a finite exposure time. The degree of this blurring is described by the speckle contrast *k*: 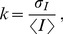
(3)


where 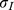 is standard deviation of the speckle intensity and 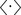 denotes averaging. The standard deviation and mean can be computed over space or time, providing better temporal or spatial resolution, respectively. Because of integration by the camera, flow speed of the scattering particles is inversely proportional to the characteristic decay time 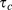 of the light intensity autocorrelation function: the faster the particles move, the smaller the 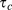. Speckle contrast value in turn is related to the ratio between the exposure time *T* and correlation time 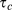: 
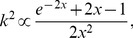
(4)


where 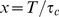. Equation 4 forms the basis of the relative blood flow measurement in laser speckle flowmetry instrumentations.

To acquire laser speckle flowmetry data we used the Moor FLPI laser speckle camera from Moor Instruments (Millwey, Axminster, UK). Moor FLPI measures tissue perfusion by performing intensity statistics analysis on images of tissue, illuminated with a 50 mW 785 nm laser light and acquired by a CCD video camera. Because we were interested in the slow dynamics (TGF rhythm), we chose to increase spatial resolution at the cost of acquisition speed by using the temporal analysis mode available in the Moor FLPI system. Using this mode, in each experiment we typically recorded 2000–2500 images with high resolution and at relatively low speed (1 frame/sec).

### Frame pre-processing

LSF frame sequences were processed offline using custom software written in the Python programming language with the use of SciPy [32] and Matplotlib [33] open source libraries. The custom software is described in [34] and is distributed under the GNU General Public License.

Frames captured by the camera had the size 568×760 pixels and were too large for pixel-wise analysis for our purposes. To reduce data size and to suppress pixel noise we applied a low-pass filter to each frame by convolution with a Gauss kernel (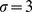 pixels) and then downsampled to 284×380 pixel images. Spatial smoothing by low-pass filtering was necessary to avoid aliasing artifacts after downsampling.

Though the camera has been staged so as to maximize the area occupied by the kidney, the frame contained small areas in the corners that were outside the kidney, and because the peritoneum covering the kidney was purposely left intact, an occasional area of fat remained within the image field. Areas outside the kidney or obscured with interfering objects were discarded from the analysis by image masking. Noting that irrelevant areas have low signal amplitude and constitute only a small portion of the frame, the following procedure was used to mask unwanted areas out. First 20 frames from the acquired frame sequence were time-averaged to produce a mean frame. Only pixels with intensity higher than 0.15 quantile of overall pixel intensity distribution in the mean frame were chosen for analysis. A threshold at 0.15 quantile is an empirical value that resulted in satisfactory kidney masks for the data set.

After spatial downsampling, the LSD time series in each pixel were normalized to their standard deviation. Normalization was done after subtraction of the time-averaged frame from all frames in the downsampled LSF frame sequence.

### Continuous wavelet transform

Time-frequency analysis was performed with continuous wavelet transform (CWT) [35]. In brief, the CWT allows one to follow the temporal variation of the magnitude, phase and frequency of the various spectral components in non-stationary time series. The continuous wavelet-transform of a signal 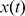 at scale *a* and time *t* is given by: 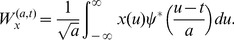
(5)


where 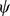 is a “mother” wavelet function. This function should be soliton-like with zero average, and the wavelet transform is obtained by a convolution of the signal with dilated and translated copies of the “mother” wavelet. We used Morlet wavelet, which is a plane wave, modulated with a Gaussian, as the “mother” wavelet: 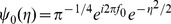
(6)


where 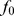 is the central frequency of the wavelet. We used 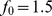 to allow for a balance between time and frequency resolution. CWT decomposition was done only in the time domain, i.e. for normalized LSF time series in each pixel. An in-house open-sourse software Swan (http://cell.biophys.msu.ru/static/swan/) was used to perform CWT. After CWT decomposition we extracted instantaneous frequency and phase values from the wavelet spectrogram 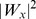 as described and illustrated in the Results section.
